# Controlling Octahedral Rotations in a Perovskite via Strain Doping

**DOI:** 10.1038/srep26491

**Published:** 2016-05-24

**Authors:** A. Herklotz, A. T. Wong, T. Meyer, M. D. Biegalski, H. N. Lee, T. Z. Ward

**Affiliations:** 1 Materials Science and Technology Division, Oak Ridge National Laboratory, Oak Ridge, TN, 37831, USA; 2 Materials Science & Engineering, University of Tennessee, Knoxville, TN 37996, USA; 3 Center for Nanophase Materials Sciences, Oak Ridge National Laboratory, Oak Ridge, TN, 37831, USA

## Abstract

The perovskite unit cell is the fundamental building block of many functional materials. The manipulation of this crystal structure is known to be of central importance to controlling many technologically promising phenomena related to superconductivity, multiferroicity, mangetoresistivity, and photovoltaics. The broad range of properties that this structure can exhibit is in part due to the centrally coordinated octahedra bond flexibility, which allows for a multitude of distortions from the ideal highly symmetric structure. However, continuous and fine manipulation of these distortions has never been possible. Here, we show that controlled insertion of He atoms into an epitaxial perovskite film can be used to finely tune the lattice symmetry by modifying the local distortions, i.e., octahedral bonding angle and length. Orthorhombic SrRuO_3_ films coherently grown on SrTiO_3_ substrates are used as a model system. Implanted He atoms are confirmed to induce out-of-plane strain, which provides the ability to controllably shift the bulk-like orthorhombically distorted phase to a tetragonal structure by shifting the oxygen octahedra rotation pattern. These results demonstrate that He implantation offers an entirely new pathway to strain engineering of perovskite-based complex oxide thin films, useful for creating new functionalities or properties in perovskite materials.

*AB*O_3_ perovskites possess some of the most technologically relevant yet least understood phenomena in condensed matter physics, such as ferroicity, metal-insulator transitions, and superconductivity. The variety of properties is based on the ability of the perovskite structure to incorporate a wide combination of *A* and *B* site ions despite large ion size differences. This structural flexibility is in part enabled by the accommodation of internal stress through structural distortions to the parent simple cubic 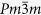 structure^1^. They can be classified into: i) tilts and rotations of the *B*O_6_ octahedra, ii) stretching of oxygen octahedra, and iii) cation displacements. In addition, perovskites are well known for their strong coupling between structural distortions and physical properties^2^. The strong coupling means that even a small change to distortions can induce a subtle effect that can have a dramatic and often unexpected impact on electronic and magnetic behaviors. The metal-oxygen-metal (or cation-anion-cation) bond angles, which directly depend on unit cell dimensions and oxygen octahedra tilting angles, have been shown to directly influence the band width and orbital occupation^3^,^4^. Thus, the ability to finely control structural distortions in the perovskite structure is a much needed capability both to functionalize these materials and to address fundamental questions regarding structure-property relationships.

Heteroepitaxial strain engineering has emerged as a powerful technique to manipulate the structure of perovskite thin films^5^,^6^,^7^,^8^,^9^,^10^. However, this common approach is severely limited by the number of available substrates that only enable tailoring of strain states in a discrete manner, which impedes our ability to finely and continuously tune lattice strain and, consequently, the resulting thin film properties. Further, due to the Poisson effect, tuning the biaxial in-plane strain of a thin film always involves modification of all three lattice directions. On the other hand, strain doping thin films by implantation of noble He atoms was recently shown to provide a means to effectively bypass the Poisson limitations, thus, allowing uniaxial lattice expansion^11^,^12^. In the present work, we use SrRuO_3_ (SRO) thin films as a model system to show that strain doping can also be used as an effective method to manipulate oxygen octahedral distortions of perovskite thin films in a continuous and reversible manner. This approach allows us to gain an unprecedented level of structural control.

SrRuO_3_ (SRO) is a 4d transition metal oxide with an orthorhombic bulk structure that is described by the Glazer notation *a*^−^*a*^−^*c*^+^^13^. This means that the oxygen octahedral rotation pattern is characterized by out-of-phase rotations of the same magnitude about the two in-plane axes and by in-phase rotations about the c axis^14^,^15^. In the bulk, SRO exhibits a structural transition from the low symmetry orthorhombic phase (O) to the high temperature tetragonal phase with *a*^*0*^*a*^*0*^*c*^−^ pattern (T) at approximately 550 °C^16^. However this structural transition is highly sensitive to lattice mismatch-driven strain effects. In SRO films coherently grown on SrTiO_3_ (001) substrates, the O phase is preserved, but due to biaxial strain effects, the crystal transition to the T phase is reduced to about 300 °C^17^,^18^,^19^,^20^. Larger compressive or tensile strain has been found to suppress this O-T phase transition below room temperature^17^,^21^,^22^,^23^,^24^,^25^,^26^. Importantly, these orthorhombic and tetragonal distortions to the cubic perovskite unit cell can be observed with lab scale 4-circle x-ray measurements. The small energetic difference and the discriminability between the two phases makes coherent SRO films grown on SrTiO_3_ an ideal model system to study the influence of the purely structural response to strain doping. It is shown that the octahedral character of the SRO lattice can be continuously controlled with He implantation and that the resultant structures allow access to geometries outside the reach of traditional, epitaxy-driven strain approaches.

## Experimental Results

A 20 nm thick film of SRO was grown on a (001) oriented single-crystalline SrTiO_3_ (STO) substrate and was then capped *ex-situ* with a 10 nm thick buffer Au film. After the deposition, He was implanted at an energy of 4 keV with a range of doses up to 2 × 10^16 ^He/cm^2^. The nobility of He assures that no extra electrons or holes are introduced into the films and doping effects are solely of structural nature. The small size of the He atoms and the low energy used minimize the damage to the perovskite lattice upon the ion bombardment. The buffer layer was used not only to further reduce the possibility of dislocation generation, but to minimize the impact of surface sputtering from the oxide film. Previous studies have shown that the energies and doses used here do not induce amorphization^27^ or induce oxygen non-stoichiometry in perovskite oxide films^11^. The penetration depth of He into the perovskite lattice is in the order of 40 nm at 4 keV^28^. When He is implanted into the perovskite lattice, it is trapped at interstitial lattice sites and causes an expansion of the unit cell. In coherent films, the in-plane lattice is epitaxially locked to the substrate. The internal stress introduced by He will therefore be relieved only by an increase of the out-of-plane lattice or a change in oxygen octahedra tilts.

**Figure 1:**
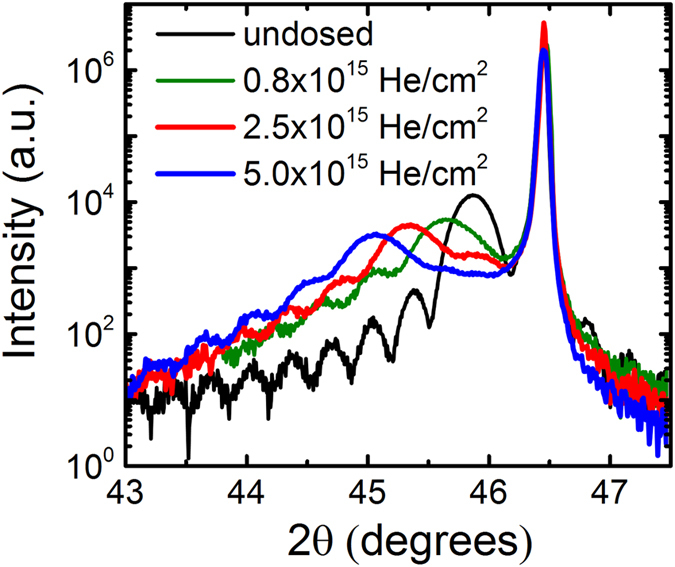
2θ-θ scans around the (002)_*pc*_ reflection of a 20 nm SrRuO_3_ film epitaxially grown on SrTiO_3_ substrate under increasing He doses. Increasing the He dose is shown to drive c-axis expansion while maintaining a high degree of strain uniformity throughout the film thickness as indicated by presence of Laue peaks even at the highest dose.

Figure 1 shows X-ray diffraction (XRD) 2θ-θ scans of the SRO films around the (002) reflection of the STO substrate before and after progressively changing the He doses. The undosed SRO film is epitaxial to the substrate with high structural quality as revealed by the clear Laue fringes. The pseudocubic out-of-plane lattice parameter of the undosed film is *c*_*pc*_ = 3.952 Å. This value is larger than the bulk pseudo-cubic lattice parameter of *c*_*pc,bulk*_ = 3.928 Å^29^,^30^ due to the Poisson effect as the film is under compressive strain on STO (*a*_*STO*_ = 3.905 Å). The coherent growth on STO is expected from many previous studies on the growth of SRO films^17^,^23^,^25^. It is worth noting that the measured out-of-plane lattice parameter of 3.952 Å is exactly what is expected of a stoichiometric SRO film fully epitaxially strained on an STO substrate given SRO’s known Poisson ratio of SRO (ʋ = 0.33)^31^. By introducing progressively higher doses of He into the SRO lattice, we observe that the 002 peak shifts to lower angles. This result indicates that the average out-of-plane lattice parameter is expanded. The out-of-plane expansion from the as-grown state is shown as 0.33% for the 0.8 × 10^15 ^He/cm^2^ low dose, 1.14% for the 2.5 × 10^15 ^He/cm^2^ moderate dose, and 1.75% for the 5 × 10^15 ^He/cm^2^ high dose. We observe that there is a slight peak broadening that accompanies an increase in He dose, which is expected due to the inherent Gaussian distribution of He in the lattice after implantation. The cap layer method described above is sufficient to greatly reduce these effects so that uniformity is still very good as evidenced by the Laue fringes and their uniform shift with the film peaks. From these data, we can clearly see that the introduction of He into the film allows control over the length of the out-of-plane parameter.

**Figure 2:**
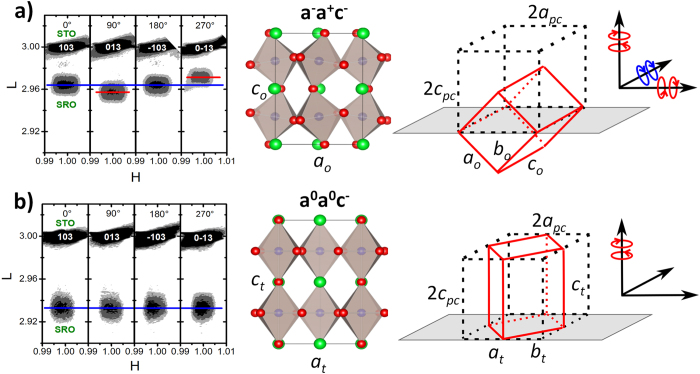
RSMs around the {103}_*pc*_ lattice reflections (left) with the corresponding side view diagrams (middle) and diagrams of the unit cell orientations (right). **(a)** The undosed film shows a clear orthorhombic distortion of the crystal structure that is in agreement with an in-plane oriented unit cell and a *a*^−^*a*^+^*c*^−^ rotation pattern. **(b)** A He dose of 2.5 × 10^15^ ions/cm^2^ is sufficient to remove orthorhombic distortions present in undosed samples and drive a tetragonal structure with an upright unit cell and *a*^*0*^*a*^*0*^*c*^−^ rotation pattern. The reciprocal-lattice units are in terms of the lattice parameter of the SrTiO_3_ substrate. The circles around the axis of the coordinate system on the right illustrate the out-of-phase (red) and in-phase (blue) octahedral rotations about the relevant cubic principle axis.

To understand how strain doping impacts orthorhombic distortions, we measure reciprocal space maps (RSM) around the STO (013) substrate reflection with the sample successively rotated by 90° with respect to the film normal. As expected, an orthorhombic distortion of the perovskite unit cell is clearly evidenced for the undosed SRO film by the different spacings of the peaks with respect to the substrate peak position (Fig. 2a). This result shows that the as-grown SRO film is epitaxial to the STO substrate with an in-plane oriented orthorhombic unit cell, which is in agreement with previous studies^17^,^18^. After implanting 2.5 × 10^15 ^He/cm^2^, we see that the orthorhombic distortion is no longer visible (Fig. 2b). The blue line highlights that the SRO peak separation to the STO substrate is identical for every reflection and that peak separation is increased as compared to that of the as-grown film due to the out-of-plane lattice expansion. This trend also reiterates the fact that the in-plane lattice remains locked to the substrate and that strain uniformity throughout the film thickness is excellent. Ultimately, these results demonstrate that the induced out-of-plane strain is sufficient to trigger a transition from an orthorhombic to a tetragonal unit cell.

**Figure 3:**
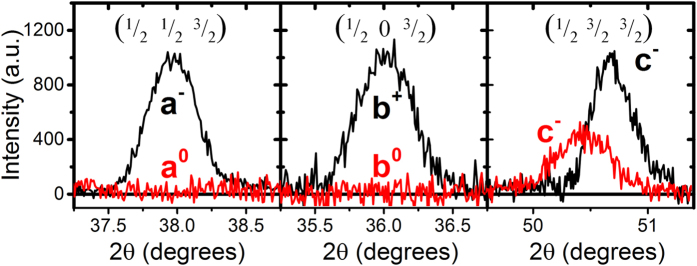
2θ-θ scans through the half-ordered (^1^/_2_^1^/_2_^3^/_2_)_pc_, (^1^/_2_ 0 ^3^/_2_)_pc_ and (^1^/_2_^1^/_2_^3^/_2_)_pc_ reflections of the undosed (black) and 5 × 10^15^ He/cm^2^ dosed (red) SRO film. The suppression of the first two peaks indicates the shift of the octahedral rotation pattern during the strain-induced O-T phase transition from *a*^−^*a*^+^*c*^−^ to *a*^*0*^*a*^*0*^*c*^−^.

However, although the tetragonality of the unit cell (*a*_*t*_ = *b*_*t*_) of the He dosed SRO films indicates a phase transition from the low-temperature orthorhombic to the high-temperature tetragonal bulk phase, direct proof of a change of the octahedral rotation pattern can only be provided by measurements that are sensitive to the internal symmetry of the crystal structure. Octahedral rotations cause a doubling of the unit cell with respect to the pseudocubic unit cell and thus, are reflected by the emergence of half-ordered XRD peaks. Synchrotron studies of half-ordered reflections on perovskite oxide thin films have been conducted recently to determine their exact octahedral rotation patterns^19^,^32^,^33^. Due to the generally low intensity of half-ordered reflections, lab-source XRD instruments are less suitable for these studies. However, the excellent structural quality of epitaxial SRO films and the rather large octahedral rotations present in orthorhombic SRO allowed us to measure half-ordered reflections with our lab-source diffractometer. Figure 3 shows 2θ-θ scans for an undosed and a 5 × 10^15 ^He/cm^2^ dosed film through the (^1^/_2_^1^/_2_^3^/_2_), (^1^/_2_ 0 ^3^/_2_), and (^1^/_2_^3^/_2_^3^/_2_) reflections that are representative of octahedral rotations about the pseudocubic *a*, *b*, and *c* axis, respectively. The undosed film shows all three half-ordered reflections, which is in agreement with the *a*^−^*a*^+^*c*^−^ rotation pattern expected for orthorhombic films grown epitaxially on SrTiO_3_ substrates^19^. The dosed film, however, lacks the (^1^/_2_^1^/_2_^3^/_2_) and (^1^/_2_ 0 ^3^/_2_) reflections. This unambiguously reveals that the octahedral rotation pattern is shifted under He implantation and rotations about the in-plane axes are diminished. The (^1^/_2_^3^/_2_^3^/_2_) reflection remains. This indicates that octahedral rotations about the out-of-plane axis persist, which is in perfect agreement with the *a*^*0*^*a*^*0*^*c*^−^ rotation pattern of the high-temperature tetragonal bulk phase. Thus, our measurements show that strain doping induces a phase transition from the orthorhombic to the tetragonal phase that is accompanied by a change of the octahedral rotation pattern.

**Figure 4:**
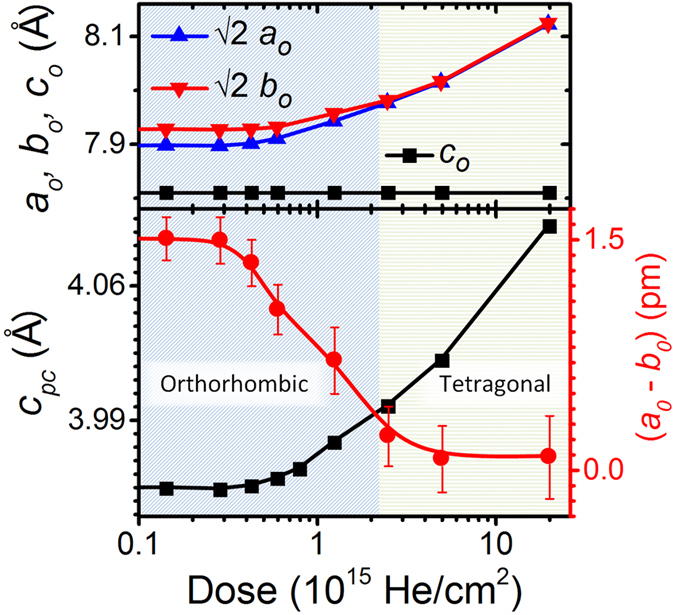
The top panel gives orthorhombic lattice parameters *a*_*o*_, *b*_*o*_, and *c*_*o*_ as a function of He dose. The orthorhombic *c* axis is clamped to the substrate and remains constant, while *a*_*o*_ and *b*_*o*_ increase upon strain doping. The bottom panel shows the evolution of the pseudocubic out-of-plane parameter *c*_*pc*_ and orthorhombic distortion (*a*_*o*_ − *b*_*o*_) of the films as a function of He dose. Increasing the He dose allows for fine and continuous control over octahedra rotation and tetragonality.

Discrete shifts from the O to T phase under biaxial in-plane strain in SRO have been previously predicted and observed, however the ability to finely control this transition has never been possible due to the inability to overcome Poisson effects and the limited number of available substrates^17^,^23^,^25^,^26^. Figure 4 shows that strain doping is a viable means to control orthorhombic distortions and drive tetragonality in a highly controllable manner. Here, we observe the impact of increasing He concentration on the pseudocubic and orthorhombic lattice parameters and the orthorhombic distortion, quantified by the difference of orthorhombic unit cell lattice parameters (*a*_*o*_ − *b*_*o*_). The orthorhombic distortion remains constant at low He doses, but quickly reduces toward zero above a He dose of 0.4 × 10^15 ^He/cm^2^, which corresponds to an out-of-plane strain of only about 0.13%. As the phase transition takes place, the orthorhombic distortion is gradually reduced until it is extinguished at doses above 3 × 10^15 ^He/cm^2^. A non-linear increase of the out-of-plane lattice parameter is also observed which indicates an increase of the unit cell volume during the O-T phase transition. This is in agreement with bulk^16^ and thin film^23^ studies. In the tetragonal phase, in-plane octahedra rotations are absent, which means that further increasing of out-of-plane strain only drives further elongation of the RuO_6_ octahedron.

**Figure 5:**
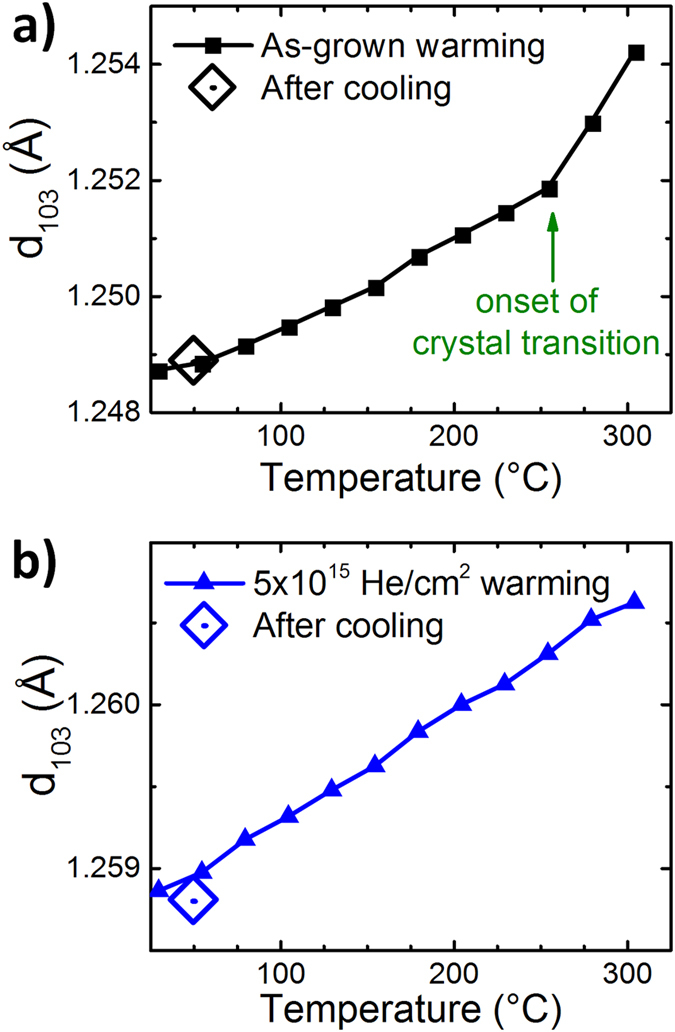
Temperature dependent change in the lattice spacing of the (103) planes during heating to 305 °C (closed symbols) and after cooling to 50 °C (open symbols). **(a)** The as-grown undosed film shows roughly linear expansion along the (103) plane during heating until the onset of the tetragonal crystal phase transition and with a return to character upon cooling. **(b)** The 5 × 10^15 ^He/cm^2^ dosed film shows linear expansion without the crystal transition signature and demonstrates a slight decrease in length along the (103) plane after cooling which can be attributed to the loss of He above ~250 °C^11^.

Due to the small atomic radius of He, it could be expected that the stability of the induced strain state could be poor. However, we find that He diffusion is negligible at ambient temperatures and that dosed films stored for at least several months showed no degradation in strain state. Figure 5 shows the effects of post-annealing in air on the structural stability for both undosed and highly dosed films by observing the (103) lattice spacing with increasing temperature. A linear increase in the lattice spacing is observed for both samples at low temperatures due to thermal expansion. The undosed film shows an upturn beginning at about 260 °C which is consistent with the temperature induced O-T phase transition previously shown to occur at 300 °C for biaxially strained films grown on SrTiO_3_ substrates^23^. The dosed film is lacking of this upturn as the O-T phase transition is already completed due to the increased out-of-plane strain. After heating the samples to 305 °C, the films were cooled down to 50 °C. The lattice parameters before and after the heating cycle are almost identical. The very slight drop in spacing of the dosed film is most likely the result of a small loss of He, which is not unexpected as previous studies showed that He in a perovskite started to evacuate above 250 °C^11^.

**Figure 6:**
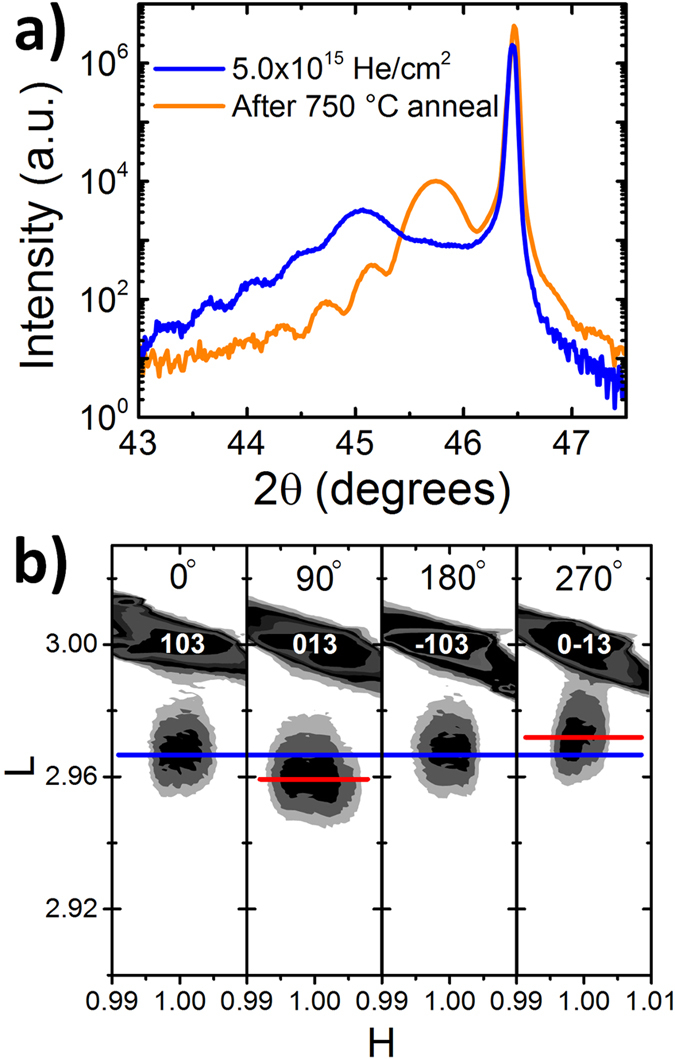
Structural characteristics of 5 × 10^15 ^He/cm^2^ dosed SRO film after annealing for 1 h at 750 °C. **(a)** XRD 2θ-θ scan around the (002)_*pc*_ reflection before and after anneal shows that the c-axis has returned toward the as-grown undosed length. **(b)** RSMs around the {103}_*pc*_ reflections after anneal demonstrates a return to orthorhombic character with octahedral tilts clearly visible. For comparison, see Fig. 2 for RSMs from the film before thermal annealing.

This high temperature instability of the He atoms in the lattice allows for additional tunability when designing a strain state as shown in Fig. 6. Here, the highly dosed 5 × 10^15 ^He/cm^2^ film was annealed for 1 hour at 750 °C. We observe that the out-of-plane lattice parameter has been reduced toward the as-grown state and that the orthorhombic distortion has returned. The *c*_*pc*_ lattice parameter and (*a*_*o*_ − *b*_*o*_) values are given as 3.967 Å and 0.0136 Å, which puts this film in a strain state not originally accessed after initial dosing. This outcome shows that strain doping can be used to progressively tune octahedra distortions by controlling implant dose and energy, and that these distortions can be modified by post-processing or even erased through a simple high temperature anneal. This observation thus opens the possibility of writing and erasing oxygen octahedra rotation patterns into epitaxial thin films by He implantation and subsequent heating steps, respectively.

## Conclusions

Epitaxial SrRuO_3_ was used as a model system to demonstrate the tailoring of oxygen octahedral distortions in perovskite oxide thin films using He implantation. A structural phase transition from the bulk-like orthorhombic phase to a tetragonal phase, which is directly related to a modification of the oxygen octahedra rotation pattern, is shown to be continuously controllable with He doping. This strain doping is found to be not only stable under ambient conditions, but reversible by thermal annealing. Our novel approach of strain engineering with He doping is of general nature and can be extended to other perovskite oxides or even other material classes, such as garnets, apatites, or spinels, where strong lattice-octahedral rotation coupling is expected^34^,^35^. These results open a new direction of tailoring oxides’ local structure and lattice symmetry with a finesse and precision that was previously inaccessible. The simplicity of this method means that it is suitable for wafer-scale processing technology and thus should be applicable to whole crystal imprinting or lithographically-directed local writing of oxygen octahedra patterns.

## Methods

A 20 nm thick film of SRO has been grown by pulsed laser deposition from a stoichiometric target. The single-crystalline SrTiO_3_ (STO) substrate was (001) oriented and TiO_2_-terminated. The growth temperature and layer energy were 750 °C and 1.5 J/cm^2^, respectively. The growth has been carried out in fairly high oxygen pressure of *p*_*O2*_ = 200 mTorr to ensure an orthorhombic structure of the as-grown film. After growth the films were annealed for 5 min and cooled in 600 mbar of oxygen. After the SRO growth the 10 nm thick buffer Au film was sputtered onto the SRO layer at room temperature. He implantation was carried out at an energy of 4 keV in a separate chamber with a base pressure of about 1 × 10^−8^ Torr using a SPECS IQE 11/35 ion source. Structural characterization and measurement of the film thickness have been carried out by a Panalytical X’Pert MRD diffractometer with Cu K_α,1_ radiation.

## Additional Information

**How to cite this article**: Herklotz, A. *et al.* Controlling Octahedral Rotations in a Perovskite via Strain Doping. *Sci. Rep.* **6**, 26491; doi: 10.1038/srep26491 (2016).
